# Time to rethink ICD indications in non-ishaemic cardiomyopathy? Evidence from a meta-analysis across therapeutic eras

**DOI:** 10.1093/eschf/xvag047

**Published:** 2026-02-10

**Authors:** Mislav Puljevic, Vedran Velagic, Hana Ivandic, Pero Hrabac, Marina Petrovic, Branimir Pervan

**Affiliations:** Cardiovascular Department, University Hospital Centre Zagreb, Kispaticeva 12, Zagreb 10000, Croatia; School of Medicine, University of Zagreb, Salata 3, Zagreb 10000, Croatia; Cardiovascular Department, University Hospital Centre Zagreb, Kispaticeva 12, Zagreb 10000, Croatia; School of Medicine, University of Zagreb, Salata 3, Zagreb 10000, Croatia; Faculty of Electrical Engineering and Computing, University of Zagreb, Unska 3, Zagreb 10000, Croatia; School of Medicine, University of Zagreb, Salata 3, Zagreb 10000, Croatia; Cardiovascular Department, University Hospital Centre Zagreb, Kispaticeva 12, Zagreb 10000, Croatia; Faculty of Electrical Engineering and Computing, University of Zagreb, Unska 3, Zagreb 10000, Croatia

**Keywords:** Non-ischaemic cardiomyopathy, Implantable cardioverter–defibrillator, Sudden cardiac death, Heart failure, Guideline-directed medical therapy, Meta-analysis

## Abstract

Implantable cardioverter–defibrillators (ICDs) reduce sudden cardiac death (SCD) in non-ischaemic cardiomyopathy (NICM), but most evidence predates comprehensive guideline-directed medical therapy (GDMT). We quantified the relative and absolute survival benefit of primary-prevention ICDs in NICM across therapeutic eras and explored how contemporary GDMT modifies absolute benefit.

We searched MEDLINE, Embase, and CENTRAL through March 2025 and included randomized controlled trials comparing prophylactic ICD implantation vs control in NICM with left ventricular ejection fraction ≤35%. Three trials (DEFINITE, SCD-HeFT NICM subgroup, and DANISH) contributed to the quantitative synthesis. ICD therapy reduced all-cause mortality (pooled hazard ratio (HR) 0.79, 95% confidence interval (CI) 0.66–0.95) and SCD (HR 0.44, 95% CI 0.28–0.70). Five-year absolute risk reduction (ARR) was 5.9% (NNT 17) in SCD-HeFT NICM and 4.4% (NNT 23) in DANISH. Under a full-GDMT scenario parameterized from pharmacological randomized controlled trials, projected baseline risk was ∼11%, yielding ARR 2.31% (NNT 43).

All contemporary ‘GDMT-era’ absolute benefit estimates are scenario-based modelling outputs. No randomized trial has evaluated ICDs on top of full modern GDMT in NICM; therefore, these results represent illustrative ranges rather than empirical estimates.

## Graphical Abstract

**Graphical Abstract:**
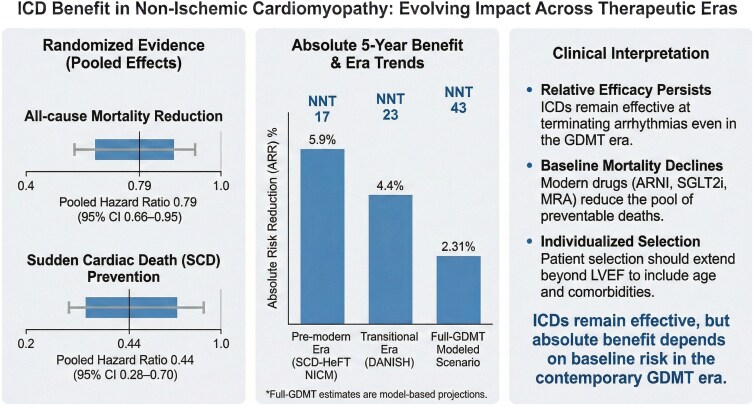

Key question: In NICM patients treated with contemporary GDMT, what is the absolute survival benefit of a primary-prevention ICD today?

Key finding: ICD therapy reduced all-cause mortality and SCD in pooled NICM trial data, but absolute benefit is highly baseline-risk dependent and may be smaller in later-era populations with lower non-arrhythmic mortality and higher CRT use; contemporary ‘GDMT-era’ estimates are illustrative modelling outputs rather than observed trial effects.

What this study adds: Because head-to-head RCTs of ICDs on top of full GDMT do not exist, we preserved randomized relative effects from legacy RCTs and applied them to trial-specific baseline risks, then projected contemporary absolute risk using effect sizes from GDMT trials (MRA, ARNI, and SGLT2i) under explicit multiplicative, independence assumptions. Observational cohorts (SwedeHF, NCDR, and CRO-INSIGHT) were used exploratorily to examine moderators (age, CRT, and era) and directional consistency—not to derive pooled treatment effects. This yields transparent, decision-supporting estimates of plausible current absolute benefit.

## Introduction

Heart failure with reduced ejection fraction (HFrEF) continues to pose a major global health challenge, affecting over 60 million people worldwide and contributing to high rates of morbidity, hospitalization, and premature death.^1^ Within this broad population, non-ischaemic cardiomyopathy (NICM) represents a particularly important subgroup. Historically, patients with NICM faced an especially high burden of sudden cardiac death (SCD), which accounted for as many as 30%–40% of all deaths in earlier cohorts.^2,3^ This high rate of arrhythmic mortality provided the rationale for the use of implantable cardioverter–defibrillators (ICDs) as a primary prevention strategy.

The first randomized controlled trial (RCT) to specifically evaluate ICD therapy in NICM was the Defibrillators in Non-Ischaemic Cardiomyopathy Treatment Evaluation (DEFINITE) trial, which randomized 458 patients with left ventricular ejection fraction (LVEF) <36% to ICD plus optimal medical therapy (OMT) or OMT alone. While ICD implantation resulted in a non-significant reduction in all-cause mortality (hazard ratio (HR) 0.65, 95% confidence interval (CI) 0.40–1.06), it significantly reduced the risk of SCD (HR 0.20, 95% CI 0.06–0.71).^4^ Shortly thereafter, the landmark Sudden Cardiac Death in Heart Failure Trial (SCD-HeFT) demonstrated that ICD therapy reduced overall mortality in a large HFrEF cohort (HR 0.77, 95% CI 0.62–0.96). Within the NICM subgroup, the effect was directionally consistent (HR 0.73, 97.5% CI 0.50–1.07), though not statistically significant due to limited power.^5^ These findings were pivotal in establishing ICDs as a class I recommendation in international guidelines for patients with symptomatic NICM, reduced LVEF, and reasonable life expectancy.^6^

Smaller RCTs also contributed but failed to alter practice due to limited sample size. The Cardiomyopathy Trial (CAT) randomized 104 patients with recent-onset dilated cardiomyopathy and showed no survival benefit of ICD over conventional therapy.^7^ Similarly, the Amiodarone Versus Implantable Cardioverter-Defibrillator-Randomized (AMIOVIRT) trial enrolled 103 patients with NICM and non-sustained ventricular tachycardia and found no difference between ICD and amiodarone with respect to overall survival.^8^ Although these studies remain historically relevant, their limited statistical power prevented them from influencing guideline recommendations. In addition, the Comparison of Medical Therapy, Pacing, and Defibrillation in Heart Failure (COMPANION) trial tested cardiac resynchronization therapy with or without defibrillation (CRT-P and CRT-D) in patients with advanced HF and conduction delay, demonstrating that CRT-D reduced mortality, although its design did not isolate the effect of ICD therapy alone.^9^

While early ICD trials established the principle that defibrillator therapy can prevent sudden arrhythmic death, the treatment landscape of HFrEF has been transformed by modern pharmacological advances. The addition of mineralocorticoid receptor antagonists (MRAs), angiotensin receptor–neprilysin inhibitors (ARNIs), and sodium–glucose cotransporter 2 (SGLT2) inhibitors has led to substantial improvements in outcomes. In EMPHASIS-HF, eplerenone reduced all-cause mortality by 24% (HR 0.76, 95% CI 0.62–0.93).^10^ In PARADIGM-HF, sacubitril/valsartan lowered all-cause mortality compared with enalapril (HR 0.84, 95% CI 0.76–0.93).^11^ More recently, DAPA-HF showed that dapagliflozin reduced all-cause mortality (HR 0.83, 95% CI 0.71–0.97),^12^ while EMPEROR-Reduced confirmed reductions in hospitalization with empagliflozin and a non-significant effect on overall survival (HR 0.92, 95% CI 0.75–1.12).^13^ Collectively, these advances have dramatically lowered baseline mortality in HFrEF populations compared with those enrolled in the early ICD trials.

Against this background, the Danish Study to Assess the Efficacy of ICDs in Patients With Non-Ischaemic Systolic Heart Failure on Mortality (DANISH) (2016) re-examined the role of ICD therapy in 1116 NICM patients treated with contemporary OMT and with a high rate of CRT use (58%). DANISH represents a transitional era before the widespread adoption of ARNI and SGLT2 inhibitors and should not be regarded as a comprehensive reference for contemporary GDMT practices. While ICD implantation halved the risk of SCD (HR 0.50, 95% CI 0.31–0.82), it did not reduce all-cause mortality (HR 0.87, 95% CI 0.68–1.12). Subgroup analyses revealed that the benefit was confined to younger patients (<68 years; HR 0.64, 95% CI 0.45–0.90), with no survival advantage in older individuals.^14^ These results highlighted the tension between relative efficacy (ICDs remain highly effective at preventing arrhythmic death) and absolute benefit (diminished when competing risks dominate). Importantly, DANISH predated routine use of ARNIs and SGLT2 inhibitors; it therefore reflects a transitional therapeutic era in which OMT and CRT penetration were high, but full contemporary GDMT was not yet achieved. Any extrapolation from DANISH to a setting of full contemporary GDMT should therefore be regarded as necessarily speculative.

This evolving evidence raises critical questions: Should every NICM patient with reduced LVEF still receive an ICD for primary prevention? To what extent might full GDMT attenuate the absolute benefit of device implantation in some subgroups? Could LVEF alone be an inadequate selection criterion in the era of personalized medicine? These uncertainties underpin the rationale for the ongoing European PROFID project, which aims to redefine ICD indications by integrating clinical, imaging, and biomarker-based risk stratification rather than relying solely on ejection fraction.^15^

The present meta-analysis was therefore designed to synthesize available randomized evidence for ICDs in NICM, compare absolute and relative benefits across therapeutic eras, and assess how contemporary pharmacological therapy modifies the risk–benefit profile of ICD implantation.

## Methods

### Search strategy and study selection

This meta-analysis was conducted according to the Preferred Reporting Items for Systematic Reviews and Meta-Analyses (PRISMA) guidelines.^16^ The study protocol was prospectively defined and registered in the PROSPERO international database (ID CRD420251162639) before data extraction and analysis.

A comprehensive literature search was performed in MEDLINE, Embase, and the Cochrane Central Register of Controlled Trials from inception through March 2025. The search terms included ‘implantable cardioverter defibrillator’, ‘ICD’, ‘non-ischaemic cardiomyopathy’, ‘dilated cardiomyopathy’, ‘sudden cardiac death’, and ‘heart failure’. Additional studies were identified through manual screening of reference lists.

The PRISMA flow diagram summarizing the search and selection process is shown in Supplementary Figure S5. In brief, the database search identified 1243 records, and manual reference screening identified 28 additional records. After the removal of 311 duplicates, 960 records remained for screening. Following title and abstract review, 42 full-text articles were assessed for eligibility. Ultimately, eight RCTs were included in the qualitative synthesis, of which three were eligible for quantitative meta-analysis of all-cause mortality and SCD (DEFINITE, SCD-HeFT NICM subgroup, and DANISH).

### Eligibility criteria

Studies were eligible if they: (i) were RCTs, (ii) enrolled patients with NICM and LVEF ≤ 35%, and (iii) compared prophylactic ICD implantation vs OMT or an active control. Trials that enrolled mixed ischaemic and non-ischaemic populations were included only if separate results for NICM were available.

The COMPANION trial was included in sensitivity analyses because it compared CRT-P and CRT-D vs OMT in patients with advanced HF, although it did not isolate the incremental effect of ICD therapy alone. Two small early RCTs, the CAT (104 patients)^7^ and the AMIOVIRT trial (103 patients),^8^ were identified but excluded *a priori* from the primary quantitative synthesis due to limited sample size and design constraints; they are reported descriptively for completeness. They are discussed narratively in the Discussion and Supplement. Major ICD trials such as MADIT-II, MUSTT, AVID, and CIDS were excluded because they enrolled ischaemic populations.

### Data extraction and quality assessment

Two reviewers independently extracted study-level data, including baseline patient characteristics, LVEF, NYHA class, medical therapy, CRT use, and outcomes (all-cause mortality, SCD). HRs with 95% CIs were extracted as the preferred summary measure. For SCD-HeFT, the prespecified NICM subgroup hazard ratio (with 97.5% CI) was used.^5^

Risk of bias was assessed using the Cochrane RoB 2 tool across five domains.^17^ Discrepancies were resolved by consensus. A summary of the risk of bias assessment is provided in Supplementary Table S4. Certainty of evidence was graded using the GRADE approach^18^ and summarized in a GRADE evidence table (Supplementary Table S5).

### Outcomes

The primary outcome was all-cause mortality. The secondary outcome was SCD, as defined in each trial. Prespecified analyses in specific patient groups examined treatment effects by therapeutic era (pre-modern vs modern), age, NYHA class, and CRT use when available.

### Statistical analysis

Meta-analysis was performed using the generic inverse-variance method. For each trial, we calculated the natural logarithm of the reported HR (log HR) and its standard error from the published HR and 95% CI; these log HRs and standard errors were then pooled using fixed-effect and random-effects (DerSimonian–Laird) models.^19^ Between-study heterogeneity was quantified using Cochran’s *Q* and *I*^2^ statistics, with *I*^2^ > 50% considered substantial.^20^ Publication bias was assessed visually with funnel plots; formal small-study tests were not performed, given the limited number of trials (*n* = 3). Random-effects models (DerSimonian–Laird with Hartung–Knapp adjustment) were used as the primary estimates, and 95% prediction intervals were reported where *k* ≥ 3. Given that heterogeneity for all-cause mortality was negligible (*I*^2^ ≈ 0%), fixed-effect results are also presented for comparison; for SCD, both fixed-effect and random-effects estimates are reported due to moderate heterogeneity. Leave-one-out sensitivity analyses were conducted to examine the influence of each study on the pooled estimate. Era-stratified analyses compared pre-modern RCTs (DEFINITE and SCD-HeFT NICM subgroup) with the transitional-era DANISH trial.

To evaluate absolute benefits, baseline risks from trial control arms were combined with the pooled ICD hazard ratio to calculate absolute risk reduction (ARR) and number needed to treat (NNT) at 5 years. Under a proportional hazards assumption, the 5-year absolute risk with an ICD was approximated as p(ICD) = HR_ICD × p(control), so that ARR = *p*(control) − p(ICD) = p(control) × (1 − HR_ICD), and NNT = 1/ARR. To model the impact of contemporary guideline-directed medical therapy (GDMT), relative risk reductions from major pharmacological RCTs—EMPHASIS-HF (eplerenone),^10^ PARADIGM-HF (sacubitril/valsartan),^11^ DAPA-HF (dapagliflozin),^12^ and EMPEROR-Reduced (empagliflozin)^13^—were applied multiplicatively to baseline mortality observed in DANISH. These GDMT-era baselines were used only for scenario-based projections of absolute benefit and were not intended to represent observed ICD effects under contemporary therapy. To test the robustness of the multiplicative independence assumption, we prespecified additional sensitivity frameworks. First, we modelled partial overlap in pharmacotherapy effects using two conservative overlap scenarios. Second, we evaluated an additive risk framework as an upper-bound scenario for combined pharmacotherapy benefit (i.e. assuming no attenuation from overlapping mechanisms). All sensitivity results are reported as ranges and summarized in Supplementary Table S1.

For scenario analyses, baseline risks from each trial were then adjusted for the relative mortality reductions observed with MRA, ARNI, and SGLT2i in their pivotal trials, assuming independence of effects, and ARRs and NNTs were recalculated using the pooled ICD effect. This multiplicative framework does not explicitly account for overlapping mechanisms of action or competing non-arrhythmic mortality. We additionally examined a competing-risk sensitivity range applying ICD benefit only to the sudden/arrhythmic component of mortality (Section 2.5.1; Supplementary Table S2). All scenario-based ARR and NNT estimates are intended as illustrative rather than definitive. For the key eras (SCD-HeFT NICM, DANISH, and the full-GDMT scenario), 95% CIs for ARR and NNT were obtained by applying the lower and upper bounds of the pooled ICD hazard ratio to the corresponding baseline risks; these uncertainty ranges are reported in *Table 3*.

Analyses were conducted in R (version 4.3.1) using the *meta* and *metafor* packages. Statistical significance was defined as *P* < .05.

Exploratory meta-regression analyses are presented in the Supplementary material and should be considered hypothesis-generating only.

#### Competing-risk sensitivity range

Because non-arrhythmic and pump-failure deaths contribute substantially to NICM mortality, we conducted a competing-risk sensitivity analysis in which the ICD effect was applied only to the sudden/arrhythmic component of mortality. We examined plausible ranges for the fraction of deaths that are sudden/arrhythmic and the ICD effect on sudden death. Results are presented as ARR/NNT ranges (Supplementary Table S2).

## Results

A total of 1243 records were identified through database searches, with 28 additional records identified by manual screening. After removal of 311 duplicates, 960 records were screened, and 42 articles underwent full-text review. Eight RCTs were included in the qualitative synthesis, of which three directly contributed to the quantitative meta-analysis (DEFINITE, SCD-HeFT NICM subgroup, and DANISH). The selection process is summarized in Supplementary Figure S5; a numerical summary is provided in *Table 1*.

**Table 1:** PRISMA study selection summary

Stage	No. of records
Records identified by database search	1243
Records identified through manual search	28
Duplicates removed	311
Titles and abstracts screened	960
Full-text articles assessed	42
RCTs included (qualitative synthesis)	8
RCTs included (quantitative synthesis)	3

### Characteristics of included studies

The trials varied in size, design, and background therapy. DEFINITE and DANISH enrolled NICM patients, while SCD-HeFT included both ischaemic and non-ischaemic populations, with a prespecified subgroup analysis available for NICM. COMPANION examined CRT-P and CRT-D in advanced HF, whereas CAT and AMIOVIRT, two small early NICM studies, were underpowered and reported neutral results. Four landmark pharmacological RCTs (EMPHASIS-HF, PARADIGM-HF, DAPA-HF, and EMPEROR-Reduced) were included for modelling the impact of contemporary therapy on baseline mortality. The key characteristics are presented in *Table 2*.

**Table 2:** Characteristics of included randomized controlled trials

Trial (year)	N (NICM%)	LVEF	Intervention vs control	CRT	FU	Main results
CAT (2002)	104 (100%)	≤35%	ICD vs OMT	0%	5 y	No survival benefit
AMIOVIRT (2003)	103 (100%)	≤35%	ICD vs amiodarone	0%	3 y	No survival benefit
DEFINITE (2004)	458 (100%)	<36%	ICD + OMT vs OMT	0%	29 mo	ACM HR 0.65 (0.40–1.06); SCD HR 0.20 (0.06–0.71)
COMPANION (2004)	1520 (≈50% NICM)	≤35%	CRT-D/CRT-P vs OMT	100%	16 mo	CRT-D HR 0.64 (0.48–0.85)
SCD-HeFT (2005)	2521 (47%)	≤35%	ICD vs amiodarone vs placebo	∼0%	45 mo	ACM HR 0.77 (0.62–0.96); NICM HR 0.73 (0.50–1.07)
DANISH (2016)	1116 (100%)	≤35%	ICD vs OMT	58%	67 mo	ACM HR 0.87 (0.68–1.12); SCD HR 0.50 (0.31–0.82)
EMPHASIS-HF (2011)	2737 (mixed)	≤35%	Eplerenone vs placebo	∼0%	21 mo	ACM HR 0.76 (0.62–0.93)
PARADIGM-HF (2014)	8442 (mixed)	≤40%	Sacubitril/valsartan vs enalapril	∼0%	27 mo	ACM HR 0.84 (0.76–0.93)
DAPA-HF (2019)	4744 (mixed)	≤40%	Dapagliflozin vs placebo	∼0%	18 mo	ACM HR 0.83 (0.71–0.97)
EMPEROR-Reduced (2020)	3730 (mixed)	≤40%	Empagliflozin vs placebo	∼0%	16 mo	ACM HR 0.92 (0.75–1.12)

NICM, non-ischaemic cardiomyopathy; LVEF, left ventricular ejection fraction; CRT, cardiac resynchronization therapy; OMT, optimal medical therapy; ACM, all-cause mortality; SCD, sudden cardiac death; HR, hazard ratio; FU, follow-up; y, years; mo, months.

### Pooled effect on all-cause mortality

**Figure 1:**
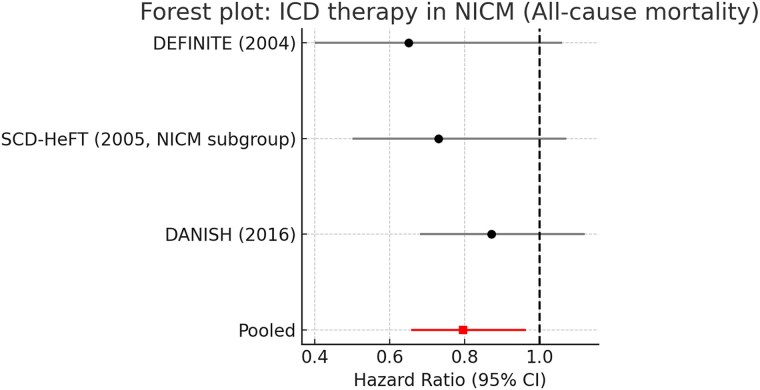
Forest plot of ICD therapy in NICM: all-cause mortality. Forest plot of randomized controlled trials evaluating implantable cardioverter–defibrillator (ICD) therapy in patients with non-ischaemic cardiomyopathy (NICM) for the study outcome of all-cause mortality. Hazard ratios (HRs) with 95% confidence intervals (CIs) are shown for DEFINITE, SCD-HeFT (NICM subgroup), and DANISH. The pooled estimate demonstrates a significant reduction in all-cause mortality with ICD implantation (HR 0.79, 95% CI 0.66–0.95), with no evidence of statistical heterogeneity (*I*^2^ = 0%). Sample sizes were 458 NICM patients in DEFINITE, 2521 patients in SCD-HeFT (47% with NICM; NICM subgroup shown), and 1116 NICM patients in DANISH

Pooling data from DEFINITE, SCD-HeFT (NICM subgroup), and DANISH demonstrated that ICD therapy significantly reduced all-cause mortality (HR 0.79, 95% CI 0.66–0.95, *P* = .014). The result was consistent across all three studies, with no evidence of statistical heterogeneity (*I*^2^ = 0%). Under random-effects modelling, the estimate was unchanged. These findings are shown in *Figure 1*.

### Pooled effect on sudden cardiac death

**Figure 2:**
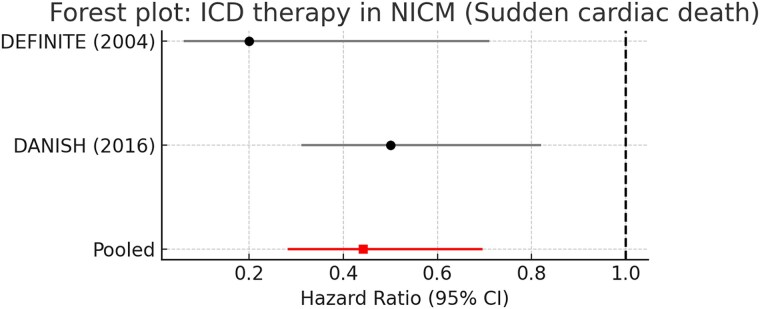
Forest plot of ICD therapy in NICM: sudden cardiac death. Forest plot of randomized controlled trials evaluating implantable cardioverter–defibrillator (ICD) therapy in patients with non-ischaemic cardiomyopathy (NICM) for the study outcome of sudden cardiac death (SCD). Hazard ratios (HRs) with 95% confidence intervals (CIs) are shown for DEFINITE and DANISH. The pooled estimate indicates a 56% relative reduction in the risk of SCD with ICD implantation (HR 0.44, 95% CI 0.28–0.70), with moderate heterogeneity attributable to differences in background therapy and CRT use. Sample sizes were 458 NICM patients in DEFINITE and 1116 NICM patients in DANISH

When restricted to trials that reported SCD as a study outcome (DEFINITE and DANISH), ICD therapy reduced the risk of arrhythmic death by more than half (pooled HR 0.44, 95% CI 0.28–0.70, *P* < .001). Moderate heterogeneity was present (*I*^2^ = 45%); random-effects modelling produced similar results (HR 0.45, 95% CI 0.27–0.74). These results are shown in *Figure 2*.

### Publication bias and sensitivity analyses

**Figure 3:**
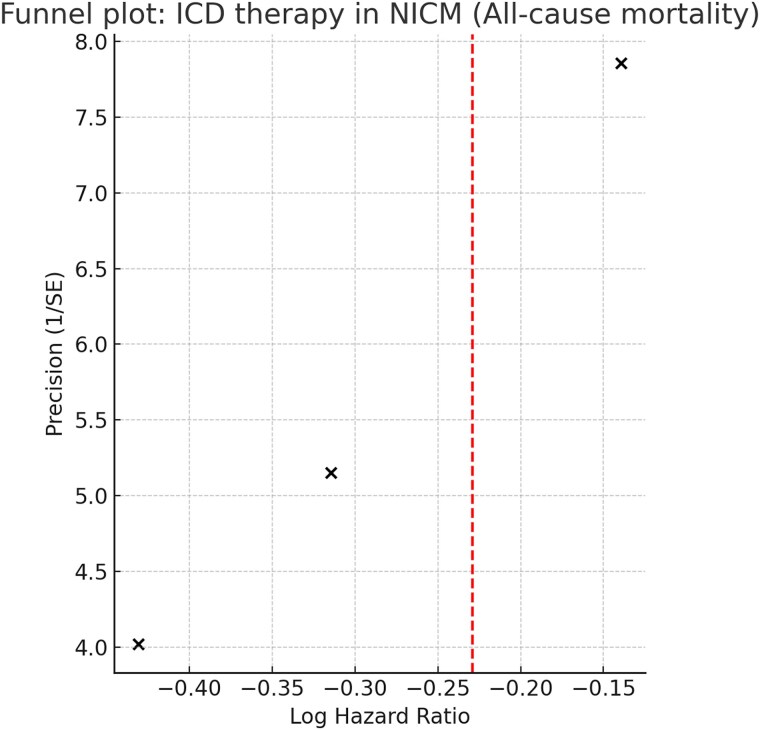
Funnel plot of ICD therapy in NICM: all-cause mortality. Funnel plot of randomized controlled trials evaluating implantable cardioverter–defibrillator (ICD) therapy in patients with non-ischaemic cardiomyopathy (NICM) for the study outcome of all-cause mortality. Each dot represents an individual trial (DEFINITE, SCD-HeFT NICM subgroup, and DANISH), plotted by log hazard ratio against precision (1/SE). The vertical dashed line represents the pooled effect estimate. No clear asymmetry is observed, suggesting the absence of publication bias, although the small number of included studies limits the reliability of this assessment

Visual inspection of the funnel plot for all-cause mortality revealed no evidence of asymmetry (*Figure 3*). However, with only three trials available, formal small-study tests were not performed due to low statistical power. Leave-one-out sensitivity analyses showed that the pooled effect was most influenced by the DANISH trial: exclusion of DANISH resulted in a stronger benefit (HR 0.70, 95% CI 0.52–0.94, *P* = .018), whereas exclusion of DEFINITE (HR 0.83, 95% CI 0.67–1.02) or SCD-HeFT NICM (HR 0.82, 95% CI 0.66–1.02) attenuated significance. Sequential exclusion of each trial did not materially change between-study heterogeneity; *I*^2^ remained negligible in all leave-one-out scenarios.

Exploratory meta-regression analyses are presented in the Supplementary material and should be considered hypothesis-generating only.

### Era-stratified analysis

**Figure 4:**
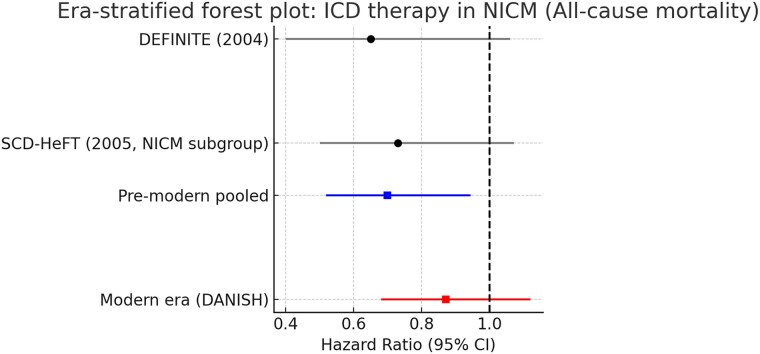
Era-stratified forest plot of ICD therapy in NICM: all-cause mortality. Era-stratified forest plot of randomized controlled trials evaluating implantable cardioverter–defibrillator (ICD) therapy in non-ischaemic cardiomyopathy (NICM) for all-cause mortality. In the pre-modern era (DEFINITE and SCD-HeFT NICM subgroup), ICD implantation was associated with a significant reduction in mortality (pooled HR 0.70, 95% CI 0.52–0.94). In contrast, in the transitional era (DANISH), conducted on a background of optimized medical therapy and high cardiac resynchronization therapy (CRT) use, ICD therapy did not reduce all-cause mortality (HR 0.87, 95% CI 0.68–1.12). These findings are consistent with a smaller absolute benefit in the context of optimized medical therapy and high CRT use. Sample sizes were 458 NICM patients in DEFINITE, 2521 patients in SCD-HeFT (47% with NICM, NICM subgroup shown), and 1116 NICM patients in DANISH

Stratification by therapeutic era revealed clear differences. In the pre-modern era (DEFINITE and SCD-HeFT NICM subgroup), ICD therapy conferred a 30% relative reduction in all-cause mortality (HR 0.70, 95% CI 0.52–0.94, *P* = .018), with no heterogeneity. In contrast, in the transitional era represented by DANISH, ICD therapy did not significantly reduce all-cause mortality (HR 0.87, 95% CI 0.68–1.12, *P* = .28). These findings, shown in *Figure 4*, highlight that the relative and absolute survival impact of ICDs is diminished in the context of optimized medical therapy and high CRT use.

### Absolute benefit and number needed to treat

Differences in baseline mortality strongly influenced the absolute benefit of ICD implantation. In the SCD-HeFT NICM subgroup, 5-year mortality in the placebo group was 28%, yielding an ARR of 5.9% (95% CI 1.4–9.5) and an NNT of 17 (95% CI 11–71). In DANISH, with a 5-year control mortality of 21%, the ARR was 4.4% (95% CI 1.1–7.1), with an NNT of 23 (95% CI 14–95).

When modelled against the additional survival gains achieved with modern pharmacological therapies, absolute benefit declined further. Incorporating eplerenone (EMPHASIS-HF) reduced baseline risk to 16.0% (NNT 30); sacubitril/valsartan (PARADIGM-HF) to 17.6% (NNT 27); dapagliflozin (DAPA-HF) to 17.4% (NNT 27); and combined MRA + ARNI + SGLT2 inhibitor therapy reduced projected baseline risk to 11.0%, with NNT 43. These contemporary estimates are modelling outputs derived from scenario assumptions and should be interpreted as illustrative ranges rather than observed trial effects. These results are detailed in *Table 3*.

**Table 3:** Absolute risk reduction and number needed to treat for ICD therapy across therapeutic eras (scenario-based estimates)

Era/source	Baseline 5-year risk	ICD HR	ARR (95% CI)	NNT (95% CI)
Pre-modern (SCD-HeFT NICM)	28%	0.79	5.9% (≈1.4–9.5)	17 (≈11–71)
Contemporary (DANISH)	21%	0.79	4.4% (≈1.1–7.1)	23 (≈14–95)
DANISH + MRA	16.0%	0.79	3.36%	30
DANISH + ARNI	17.6%	0.79	3.70%	27
DANISH + SGLT2i	17.4%	0.79	3.65%	27
DANISH + MRA + ARNI	13.4%	0.79	2.81%	36
DANISH + ARNI + SGLT2i	14.6%	0.79	3.07%	33
DANISH + MRA + ARNI + SGLT2i	11.1%	0.79	2.33% (≈0.6–3.8)	43 (≈27–179)

Sensitivity analyses addressing non-independence and alternative combination frameworks yielded broadly similar conclusions, with projected ARR ranging from 1.68% to 3.19% and NNT from 31 to 60 across prespecified scenarios (Supplementary Table S1). Under competing-risk sensitivity assumptions, projected absolute benefit was smaller and varied materially with the assumed sudden-death fraction and the ICD effect on sudden death: ARR ranged from 0.88% to 2.31% and NNT from 44 to 114 (Supplementary Table S2). Using a central illustrative scenario (sudden-death fraction 0.25 and HR for sudden death 0.50), ARR was 1.38% (NNT 73).

In threshold analyses, when baseline 5-year mortality was 4%, the projected ARR was 0.84% under the all-cause HR approach and 0.50% under a competing-risk example (π = 0.25, HR_SCD = 0.50), corresponding to NNT >100 (Supplementary Table S3). Across baseline 5-year mortality of 6%–12%, projected ARR remained ≥1% (Supplementary Table S3).

## Discussion

The present meta-analysis highlights both the enduring power and the diminishing returns of implantable ICDs in NICM. Across three major RCTs, ICD therapy consistently reduced the risk of SCD by more than 50%. This is a large and biologically plausible effect, consistent with the mechanism of ICDs in terminating lethal ventricular arrhythmias. Yet, despite this dramatic relative efficacy, the impact on all-cause mortality has steadily weakened. In DEFINITE and SCD-HeFT, conducted in the pre-modern era of heart failure management, ICD implantation translated into a clear survival benefit, with numbers needed to treat as low as 17. In DANISH, where nearly all patients received beta-blockers and renin–angiotensin system inhibitors and more than half had CRT, the same relative effect yielded only modest absolute benefit, with an NNT of 23. When projected onto the background of comprehensive GDMT with mineralocorticoid receptor antagonists, sacubitril/valsartan, and SGLT2 inhibitors, the NNT rises beyond 40. Thus, while ICDs remain highly effective in relative terms, the absolute survival gain in contemporary cohorts is smaller and concentrated in selected patient groups. As heart failure therapy continues to evolve—with emerging drug classes and more consistent real-world optimization of care—baseline mortality in NICM is likely to decline further, potentially attenuating the absolute survival benefit of ICDs beyond the ranges captured by our current scenarios.

Within this framework, the DANISH trial occupies a transitional position between legacy ICD trials and the era of full contemporary GDMT. Almost all patients in DANISH received beta-blockers and renin–angiotensin system inhibitors, many were treated with mineralocorticoid receptor antagonists, and more than half had CRT, yet angiotensin receptor–neprilysin inhibitors and SGLT2 inhibitors were not part of routine care at the time. DANISH therefore represents a more advanced therapeutic era than DEFINITE and SCD-HeFT, but still falls short of today’s comprehensive GDMT. Our projections for ‘full GDMT’ are extrapolations based on effect sizes from separate pharmacological RCTs and are presented as speculative quantitative scenarios rather than direct trial evidence. Accordingly, projections labelled as ‘full GDMT’ extend beyond observed DANISH-era therapy patterns and are presented as illustrative scenario-based estimates.

This paradox does not diminish the efficacy of ICDs; rather, it reflects the success of modern pharmacotherapy. As sudden arrhythmic death has declined, competing risks have emerged. Patients with NICM today are more likely to die from progressive pump failure, infection, malignancy, or comorbid conditions than from a shockable arrhythmia. This shift explains why the DANISH trial failed to show a survival benefit despite halving arrhythmic deaths, and why absolute benefit is shrinking in contemporary cohorts. It also exposes the limits of relying on LVEF as the sole gatekeeper for device therapy. An EF of 30% may no longer carry the same risk in 2025 as it did in 2005.

Device-related harms and ICD therapies varied across pivotal trials and must be weighed against absolute benefit, particularly under contemporary scenario projections with higher NNT. A structured summary of acute/procedural complications, longer-term device complications, and ICD therapies/shocks as reported in SCD-HeFT, DEFINITE, and DANISH is provided in *Table 4*.

**Table 4:** Device-related complications and ICD therapies in pivotal trials

Trial	Follow-up	Implantation/early procedural complications	Lead-related complications/revision	Device infection (any)	Serious device infection	Pneumothorax	Bleeding requiring intervention	Device removal/deactivation	ICD shocks/therapies (selected)
DEFINITE	Mean 29 months	3/229 (1.3%): 1 hemothorax, 1 pneumothorax, 1 cardiac tamponade; no procedure-related deaths	6/229 (2.6%): lead dislodgement or lead fracture (during follow-up)	1/229 (0.4%) (during follow-up)	Not reported	1/229 (0.4%) (peri-implant)	Not reported	Not reported	Reported; detailed rates not uniformly presented in the main text (trial documents reported appropriate and inappropriate shocks)
SCD-HeFT	∼5 years	Implant declined 17/829 (2.0%); implantation unsuccessful 1/829 (<0.1%); clinically significant ICD complications 5% at implantation	Reported within ‘clinically significant ICD complications’	Reported within ‘clinically significant ICD complications’	Not reported	Reported within ‘clinically significant ICD complications’	Reported within ‘clinically significant ICD complications’	32/829 (3.9%) removed during follow-up	Any shocks: 259/829 (31%); shocks for rapid VT/VF: 177/829 (21%); average annual shock rate 7.5%; average annual appropriate shock rate 5.1%
DANISH	Median 67.6 months	Reported in trial complication table (not uniformly broken down across all categories)	Reported in trial complication table (not uniformly broken down across all categories)	27/556 (4.9%)	15/556 (2.7%)	11/556 (2.0%)	1/556 (0.2%)	Not reported	Inappropriate shocks: 33/556 (5.9%)

Definitions, ascertainment, and reporting windows for device complications differed across trials; values are presented as a structured quantitative narrative summary rather than a pooled meta-analysis.

The heterogeneity of benefit across subgroups adds another layer of complexity. Younger patients clearly benefit, while older, frailer individuals do not. Patients in NYHA class II in SCD-HeFT derived substantial survival gains, whereas those in class III did not. In DANISH, patients already receiving CRT experienced no incremental benefit from an ICD. These findings converge on one principle: ICD therapy in NICM is not one-size-fits-all and should be individualized.

Observational evidence reinforces this conclusion. The Swedish Heart Failure Registry (SwedeHF)^21^ and U.S. NCDR^22,23^ data confirm that ICD implantation is associated with better outcomes in real-world NICM populations, but the absolute benefit is modest, and strongly influenced by age, sex, and comorbidity. Danish national registry follow-up of DANISH patients made the same point: benefit was confined to those younger than 70. Data from the CRO-INSIGHT study,^24^ though from a single high-volume centre, echo these international findings: among Croatian patients with NICM, survival gains from ICDs are minimal in the era of full GDMT. Together, these registries remind us that RCT findings are not merely historical curiosities—they continue to hold, but their context has changed.

The clinical implications are profound. ICDs still prevent arrhythmic deaths, but at a price: infections, lead failure, inappropriate shocks, and repeat procedures remain important harms. Beyond procedural and lead-related complications, ICD shocks and device revisions can substantially affect quality of life through anxiety, shock-related distress, and repeated hospital visits, particularly when the absolute survival gain is small. In this context, a uniform strategy of implanting ICDs in all patients with an EF ≤ 35% may no longer be appropriate for every NICM patient receiving contemporary GDMT. Instead, selection should increasingly be refined, integrating age, comorbidity burden, CRT status, competing non-arrhythmic risks, and response to GDMT. These trade-offs between modest absolute survival benefit and device-related harms should be explicitly addressed in shared decision-making, incorporating patient values, life expectancy, and tolerance for shocks and re-interventions.

The future may hold even more radical transformations. Artificial intelligence applied to electrocardiographic signals, echocardiographic strain patterns, or continuous device telemetry could uncover hidden risk profiles invisible to conventional clinical tools. Genetic discoveries may soon identify patients with arrhythmogenic mutations for whom ICDs are essential, while sparing others. One can imagine a not-so-distant era in which the decision to implant an ICD is not dictated by a single number, such as EF, but by a composite algorithm that integrates age, comorbidity, pharmacological response, imaging features, and AI-derived electrophysiological risk.

Such approaches are under active investigation, but further work is required before they can be routinely implemented in clinical practice.

Our analysis has limitations that may temper interpretation. Only three large RCTs directly inform ICD efficacy in NICM, and two small early trials (CAT and AMIOVIRT) were underpowered. Subgroup data were extracted at the trial level rather than from individual patient datasets. Modelled absolute benefits are illustrative, based on multiplicative application of relative risk reductions from pharmacological trials, and may overestimate the true effect of combined therapies. Device-related complications were not consistently reported across trials and could not be meta-analysed. Nonetheless, the consistency of relative effects, the alignment with registry data, and the era-stratified absolute risk estimates all converge on a single conclusion.

ICDs are neither obsolete nor universally indicated. They remain lifesaving in selected patients—especially younger individuals, those with high arrhythmic burden, or genetic and structural substrates of risk. But in patients fully stabilized on modern GDMT, with advanced age or major competing comorbidities, ICD implantation may provide only limited survival advantage.

The exploratory trial-level meta-regression adds context but must be interpreted with great caution. Only three adequately powered NICM RCTs were available, and each model could include only a single moderator. Higher CRT uptake, older age, and later year of publication were consistently associated with attenuation of the ICD effect, but none of these associations reached statistical significance. Given the small number of trials and the ecological nature of study-level analyses, these findings are statistically underpowered and should be regarded strictly as hypothesis-generating rather than confirmatory; a more definitive assessment will require individual patient-level data and competing-risk frameworks.

Take-home message: The question is no longer whether ICDs work in NICM-they do. The question is for whom they still matter. The future of device therapy lies in precision: choosing the right patient, at the right time, guided not only by EF but also by a deeper understanding of arrhythmic risk, and perhaps soon by the tools of artificial intelligence. As absolute survival gains diminish in contemporary practice, device-related harms (infections, lead failure, and inappropriate shocks) weigh more heavily in net clinical benefit considerations and shared decision-making.

### Limitations

**Figure 5:**
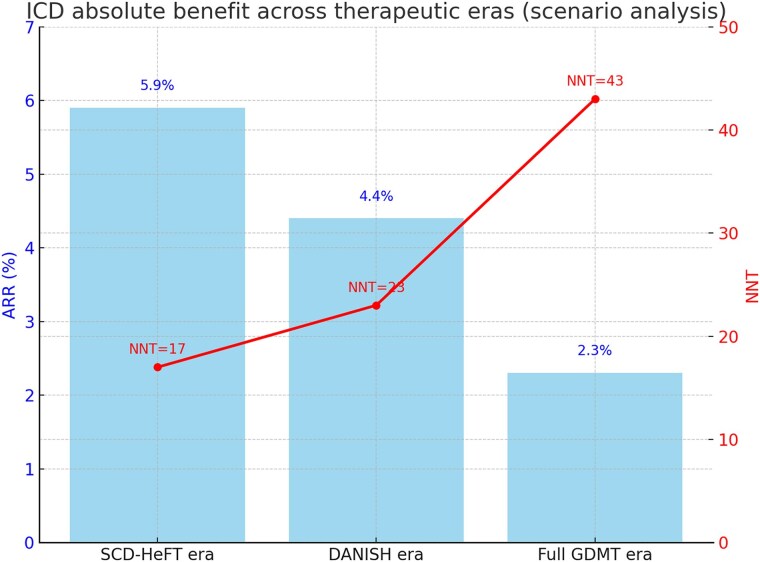
ICD absolute benefit across therapeutic eras (scenario analysis): absolute risk reduction (ARR, blue bars, shown as percentage points) and number needed to treat (NNT, red line) with ICD implantation across therapeutic eras. ARR was 5.9% (NNT 17) in the pre-modern era (SCD-HeFT), 4.4% (NNT 23) in the DANISH era, and 2.3% (NNT 43) when full contemporary GDMT (MRA, ARNI, and SGLT2i) was modelled. Values are illustrative and derived from scenario analyses applying pooled ICD hazard ratios to era-specific baseline risks; corresponding 95% confidence intervals for ARR and NNT in these eras are provided in Table 3

This meta-analysis has several limitations that should be acknowledged. First, only three adequately powered RCTs (DEFINITE, SCD-HeFT NICM subgroup, and DANISH) directly evaluated ICD therapy in patients with NICM. Two smaller early trials, CAT and AMIOVIRT, were identified but were underpowered and not included in the quantitative synthesis. Quantitatively, the two excluded trials (CAT *n* = 104; AMIOVIRT *n* = 103) together represent <10% of the total randomized evidence base (>2600 NICM participants across DEFINITE, SCD-HeFT NICM, and DANISH). Their omission therefore has negligible influence on the pooled HR, which is dominated by the adequately powered trials. As a result, the trial-level evidence base is narrower than for ischaemic cardiomyopathy. Second, subgroup data—for age, NYHA class, and CRT use—were extracted from published reports rather than individual patient datasets. While these subgroup findings are clinically compelling, they should be interpreted with caution, as they are subject to ecological bias and reduced statistical power and cannot fully adjust for patient-level covariates or interactions. Third, the ARR and NNT estimates were derived by applying pooled hazard ratios to baseline risks observed in historical and contemporary control groups. Our modelling of modern GDMT effects relied on multiplicative application of hazard ratios from pharmacological RCTs (EMPHASIS-HF, PARADIGM-HF, DAPA-HF, and EMPEROR-Reduced). Because of overlapping mechanisms, these combined estimates may overstate the true reductions in baseline risk, and thus the modelled NNTs should be regarded as illustrative rather than definitive. Importantly, contemporary GDMT trials included very low ICD uptake and did not uniformly adhere to device-therapy guidelines; accordingly, we did not use those trials to infer ICD efficacy nor to compare eras head-to-head. Instead, we preserved randomized relative effects from legacy ICD RCTs and used GDMT trial data only to parameterize background risk in scenario-based estimates—intended as decision-supporting, not predictive. Our scenario modelling assumed multiplicative and independent effects of pharmacologic therapies and ICDs and should be interpreted as illustrative rather than predictive (*Figure 5*). Where observational cohorts were referenced, they were used exploratorily to assess moderators and directional consistency and were not pooled with RCTs to derive treatment effects. Small early RCTs (CAT and AMIOVIRT) and large registries were deliberately used in a descriptive, contextual manner and were not pooled with the core RCTs to derive ICD treatment effects, to avoid mixing underpowered or observational evidence into the primary estimates. Alternative approaches, such as additive models of risk reduction, Bayesian frameworks, or formal competing-risk analyses, could more explicitly accommodate overlapping mechanisms and non-arrhythmic mortality, but would require access to individual patient-level data and were beyond the scope of this study. Fourth, device-related complications—including infections, lead failure, and inappropriate shocks—were not consistently reported across ICD trials and therefore could not be meta-analysedand patient-reported outcomes (including quality of life and psychological impact of shocks) were rarely and heterogeneously captured; as a result, the net clinical benefit may be less favourable than suggested by survival endpoints alone. These outcomes are important in the real-world balance of benefit and harm and may further reduce the net clinical benefit when absolute survival gain is small. Finally, although our funnel plot did not suggest publication bias, the small number of trials (*n* = 3) precludes a reliable statistical assessment of small-study effects. Future analyses will require integration of individual patient-level data and prospective randomized trials designed in the era of full GDMT, such as the ongoing PROFID project, to provide definitive clarity.

### Clinical implications

This study offers several novel contributions with direct clinical relevance. To the best of our knowledge, this is the first to combine evidence from randomized ICD trials with modelling of complete contemporary GDMT, including ARNI and SGLT2 inhibitors, thereby quantifying how modern therapy reduces the absolute benefit and increases the NNT for ICD implantation. In addition, by synthesizing trial results with data from large registries (SwedeHF, NCDR, and CRO-INSIGHT), it demonstrates that this attenuation is consistently observed across both randomized and real-world settings. Together, these findings emphasize that ICDs remain effective, but that careful patient selection is crucial in the current therapeutic era.

## Conclusion

ICDs reduce sudden arrhythmic death in NICM, but improvements in baseline prognosis under contemporary GDMT are expected to lower absolute survival gains. In earlier trials, the NNT to prevent one death was ∼17, whereas under plausible modern GDMT scenarios, the projected NNT is closer to 40–60, depending on assumptions. These contemporary estimates are scenario-based modelling outputs rather than observed trial effects, and no randomized trial has evaluated ICDs on top of full modern GDMT in NICM. Accordingly, the projections should be interpreted as illustrative ranges and not as practice-changing evidence. Future work should prioritize improved risk stratification and prospective evaluation to identify NICM patients most likely to derive clinically meaningful net benefit, while minimizing unnecessary device implantation in those with low expected absolute gain. These estimates may support consideration of more individualized ICD selection in NICM, but they do not justify practice-changing conclusions in the absence of randomized evidence on top of full modern GDMT.

## Supplementary Material

xvag047_Supplementary_Data

## Data Availability

No new data were generated or analysed in support of this research.
