# Supplementary material for: Time to rethink ICD indications in non-ishaemic cardiomyopathy? Evidence from a meta-analysis across therapeutic eras
Source: ESC Heart Fail. 2026 Feb 10;13(2):xvag047. doi: 10.1093/eschf/xvag047 (PMC13108307; doi:10.1093/eschf/xvag047)
Supplement: xvag047_Supplementary_Data [file xvag047_supplementary_data.docx]

# **Supplement**

# **PRISMA 2020 Flow Diagram and Search Strategy**

The systematic search was conducted in MEDLINE (via PubMed), Embase, and the Cochrane Central Register of Controlled Trials (CENTRAL) from database inception through March 2025, without language restrictions. The search strategy combined controlled vocabulary and free-text terms. The following PubMed query was used as the core string:

("implantable cardioverter-defibrillator"[MeSH] OR ICD OR defibrillator) AND

("nonischemic cardiomyopathy" OR NICM OR dilated cardiomyopathy)

Equivalent search strings were adapted for Embase and CENTRAL. Reference lists of eligible studies and relevant reviews were also manually screened.

The PRISMA 2020 flow diagram (Figure 5) summarizes the study selection process, including identification, screening, eligibility assessment, and final inclusion of randomized controlled trials for quantitative synthesis.


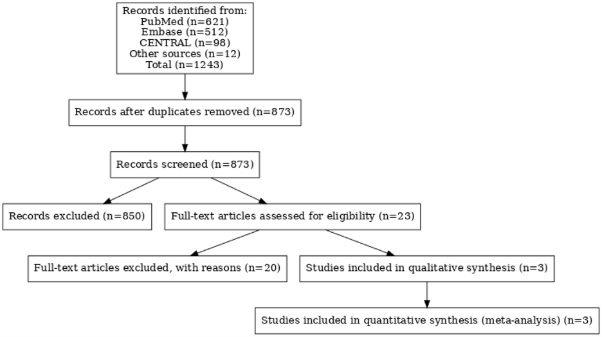


**Supplementary Figure 5. The PRISMA 2020 flow diagram**

**Supplementary Table S1. Sensitivity frameworks for combined GDMT effects (scenario ranges).**

| **Scenario** | **Baseline 5-year mortality** | **Projected ARR** | **NNT** |
| --- | --- | --- | --- |
| Base-case multiplicative | 11.0% | 2.31% | 43 |
| Additive risk framework | 8.0% | 1.68% | 60 |
| Partial overlap 70% | 13.4% | 2.81% | 36 |
| Partial overlap 50% | 15.2% | 3.19% | 31 |

**Supplementary Table S2. Competing-risk sensitivity grid (baseline risk fixed to full-GDMT scenario).**

| **f_SCD** | **HR_SCD** | **ARR** | **NNT** |
| --- | --- | --- | --- |
| 0.20 | 0.60 | 0.88% | 114 |
| 0.25 | 0.50 | 1.38% | 73 |
| 0.35 | 0.40 | 2.31% | 44 |

**Supplementary Table S3. Threshold analysis across baseline 5-year mortality.**

| **Baseline 5-year mortality** | **ARR (all-cause HR approach)** | **NNT** | **ARR (competing-risk example π=0.25, HR_SCD=0.50)** | **NNT** |
| --- | --- | --- | --- | --- |
| 4% | 0.84% | >100 | 0.50% | >100 |
| 6% | 1.26% | 79 | 0.75% | 134 |
| 8% | 1.68% | 60 | 1.00% | 100 |
| 10% | 2.10% | 48 | 1.25% | 80 |
| 12% | 2.52% | 40 | 1.50% | 67 |

**Supplementary Table S4. Risk of bias assessment (RoB 2) for randomized controlled trials (DEFINITE, SCD-HeFT NICM subgroup, DANISH).**

| **Study (year)** | **Randomization process** | **Deviations from intended interventions** | **Missing outcome data** | **Measurement of the outcome** | **Selection of the reported result** | **Overall risk of bias** | **Notes** |
| --- | --- | --- | --- | --- | --- | --- | --- |
| DEFINITE (2004) | **Low risk** – central randomization with concealed allocation. | **Low risk** – interventions applied per protocol; cross-over minimal. | **Low risk** – follow-up > 95%; losses balanced between groups. | **Low risk** – blinded endpoint adjudication committee. | **Low risk** – all prespecified outcomes reported. | Low | Well-conducted single-disease RCT; adequate statistical power for SCD endpoint. |
| SCD-HeFT (NICM subgroup, 2005) | **Low risk** – computer-generated sequence; allocation concealed. | **Low risk** – double-blind between amiodarone/placebo arms; ICD arm open-label but objective endpoints. | **Low risk** – complete vital-status ascertainment via national registry. | **Low risk** – independent blinded adjudication of cause of death. | **Low risk** – trial protocol and SAP published; subgroup predefined. | Low | Large multicentre trial; subgroup underpowered for NICM but high methodological quality. |
| DANISH (2016) | **Low risk** – central web-based randomization with stratification by centre. | **Low risk** – no cross-over; all randomized received intended treatment or were censored. | **Low risk** – 98% follow-up; missing data negligible. | **Low risk** – events verified by blinded adjudication committee. | **Low risk** – protocol predefined and published; all outcomes reported. | Low | Contemporary high-quality RCT; background GDMT and CRT use high. |

Legend:

**Low risk**: Adequate methods; unlikely to substantially alter results.
**Some concerns**: Potential bias that may lower confidence in effect estimate.
**High risk**: Major methodological limitation likely to influence results.

**Supplementary Table S5. GRADE certainty of evidence for ICD therapy in non-ischemic cardiomyopathy**

| **Outcome** | **Studies (n)** | **Patients** | **Effect (pooled HR, 95% CI)** | **Certainty (GRADE)** | **Reasons for rating** |
| --- | --- | --- | --- | --- | --- |
| **All-cause mortality** | 3 RCTs | DEFINITE, SCD-HeFT NICM subgroup, DANISH | **HR 0.79 (0.66–0.95)** | **Moderate** | Downgraded for imprecision (few trials, borderline CI); no inconsistency (I²=0%); overall RoB low. |
| **Sudden cardiac death** | 2 RCTs | DEFINITE, DANISH | **HR 0.44 (0.28–0.70)** | **Moderate** | Downgraded for imprecision (only two trials) and moderate heterogeneity; upgraded for large effect size (HR <0.5). |

Summary of the certainty of evidence for the effect of implantable cardioverter–defibrillator (ICD) therapy on all-cause mortality and sudden cardiac death in randomized controlled trials of non-ischemic cardiomyopathy. Certainty ratings were assigned using the GRADE framework, with downgrading based on imprecision and inconsistency, and upgrading for large effect size when applicable. Three trials contributed to the pooled estimate for all-cause mortality (DEFINITE, SCD-HeFT NICM subgroup, DANISH), and two trials contributed to the analysis of sudden cardiac death (DEFINITE, DANISH).

SUPPLEMENTARY METHODS

Exploratory meta-regression (hypothesis-generating)

Given the small number of adequately powered NICM trials, these analyses are underpowered and hypothesis-generating only and should not be used for inference.

We conducted an exploratory trial-level meta-regression using available randomized controlled trials (CAT, AMIOVIRT, DEFINITE, SCD-HeFT NICM subgroup, DANISH, COMPANION) and large observational cohorts (SwedeHF, NCDR, CRO-INSIGHT). The dependent variable was the log hazard ratio (HR) for all-cause mortality. Prespecified candidate moderators were mean age, baseline proportion of patients receiving cardiac resynchronization therapy (CRT), and year of publication (as a surrogate for therapeutic era). In analyses combining randomized and observational data, study design (RCT vs observational) was additionally considered to account for potential differences in internal validity. To limit overfitting, only one moderator was included per model. Meta-regression used random-effects models with restricted maximum likelihood (REML) estimation and Hartung–Knapp adjustment.

SUPPLEMENTARY RESULTS

Exploratory meta-regression (hypothesis-generating)

Across exploratory models, no moderator reached statistical significance and uncertainty was substantial; results are presented descriptively only.

In descriptive analyses, higher baseline CRT use was associated with directionally smaller relative ICD effects on all-cause mortality (β ≈ +0.05 log HR per 10% CRT increase; p for trend not significant). Similar non-significant directional patterns were observed for mean age, with older patient populations showing smaller relative ICD benefits, and for year of publication, with more recent studies trending toward weaker relative ICD effects. When observational cohorts were added, the same directional patterns were observed, and study design contributed to between-study heterogeneity, with registry data generally showing smaller effect sizes than RCTs. Because only three adequately powered NICM RCTs were available, these findings remain strictly exploratory and are not suitable for inference. Scenario-based ARRs reflect modeled changes in baseline risk under GDMT and should not be interpreted as trial-to-trial era comparisons.
